# Fc-Silenced CD89 × EGFR Bispecific Antibodies Promote Neutrophil and Macrophage Antitumor Activity

**DOI:** 10.3390/antib15040066

**Published:** 2026-07-29

**Authors:** Felix Meiser, Julia Harwardt, Christoph Hahn, Marta Lustig, Thomas Valerius, Harald Kolmar

**Affiliations:** 1 Institute for Organic Chemistry and Biochemistry, Technical University of Darmstadt, 64287 Darmstadt, Germany; felix.meiser@tu-darmstadt.de (F.M.); julia.harwardt@merckgroup.com (J.H.); 2 Division of Stem Cell Transplantation and Cellular Immunotherapy, Christian-Albrechts-University, 24105 Kiel, Germany; stu221709@mail.uni-kiel.de (C.H.); marta.lustig@uksh.de (M.L.); t.valerius@med2.uni-kiel.de (T.V.); 3 Centre for Synthetic Biology, Technical University of Darmstadt, 64287 Darmstadt, Germany

**Keywords:** CD89 (FcαRI), EGFR targeting, myeloid cell engagement, neutrophil-mediated cytotoxicity, antibody-dependent cellular phagocytosis (ADCP), Fc silencing (LALA), immune cell engagers

## Abstract

Background/Objectives: CD89 (FcaRI), the receptor for immunoglobulin A (IgA), is expressed on myeloid cells, including neutrophils, monocytes, and macrophages. It is known to mediate potent effector functions against tumor cells, but the clinical development of IgA-based therapeutics is hindered by manufacturing challenges and IgA’s short plasma half-life. Methods: Using yeast display, we generated EGFR × CD89 bispecific common light chain antibodies and investigated their biophysical properties, neutrophil-mediated cytotoxicity and macrophage phagocytosis in vitro. Results: CD89-targeting constructs induced potent neutrophil cytotoxicity and macrophage phagocytosis in vitro. A symmetric 2 + 2 IgG1 Fc-silenced variant showed the most consistent potency across neutrophil and macrophage effector functions. Fc silencing enhanced neutrophil ADCC and did not induce detectable neutrophil or PBMC fratricide under the conditions tested, suggesting a limited propensity for off-target immune cell killing. Conclusions: These findings support further preclinical evaluation of CD89-targeting bispecific antibodies and indicate that Fc silencing may differentially shape neutrophil- and macrophage-mediated antitumor activity.

## 1. Introduction

Immune cell engagers, particularly T cell-redirecting bispecific antibodies, have transformed the treatment of hematological cancers. However, translating this success to solid tumors has proven challenging due to poor T cell infiltration, tumor heterogeneity, and a highly immunosuppressive tumor microenvironment (TME) [1]. Within the TME, tumor-associated macrophages (TAMs) and neutrophils (TANs) are recruited and contribute to immune suppression by secreting anti-inflammatory cytokines such as TGF-β and IL-10, as well as promoting tumor angiogenesis and metastasis [2,3]. Despite these immunosuppressive functions, TAMs and TANs retain functional plasticity and can be activated to elicit potent antitumor responses, representing a promising avenue for immunotherapeutic intervention [4,5].

While IgG-based approaches that engage Fcγ receptors (FcγRs) potently activate macrophages, their efficacy in neutrophils is limited. This is, in part, due to the high expression of CD16b (FcγRIIIb) on neutrophils, a glycosylphosphatidylinositol-anchored decoy receptor that sequesters IgG and impairs antibody-dependent cellular cytotoxicity (ADCC) [6]. Consequently, therapeutic strategies have turned to CD89, which mediates potent effector functions of IgA in both neutrophils and macrophages. In neutrophils, IgA-driven cytotoxicity is primarily mediated by trogocytosis—a mechanical process in which tumor cell membranes are disrupted, ultimately leading to trogoptosis [7]. Additionally, CD89 engagement in neutrophils triggers secretion of leukotriene B4 (LTB4), driving neutrophil swarming and amplifying local immune responses [8,9]. In macrophages, it can induce phagocytosis of cancer cells and is reported to enhance CD8^+^ T cell infiltration via antigen presentation [10].

Despite these functional advantages, IgA-based therapeutics have seen limited clinical adoption because of manufacturing challenges and rapid clearance due to the absence of neonatal Fc receptor (FcRn)-mediated recycling [11]. Engineering approaches, such as tandem IgA–IgG formats and stabilized IgA2.0, have improved serum stability and manufacturability and have demonstrated the efficacy of targeting myeloid cells in solid tumor immunotherapy [12,13]. An alternative strategy involves the use of Fab-based CD89 binders within an IgG1 backbone, enabling precise control of affinity, valency, and Fc engineering. One such example is TrisomAb [14], a bispecific Fc-competent IgG1 antibody targeting CD89 and the Epidermal growth factor receptor (EGFR), that was generated by controlled Fab arm exchange [15].

**Figure 1:**
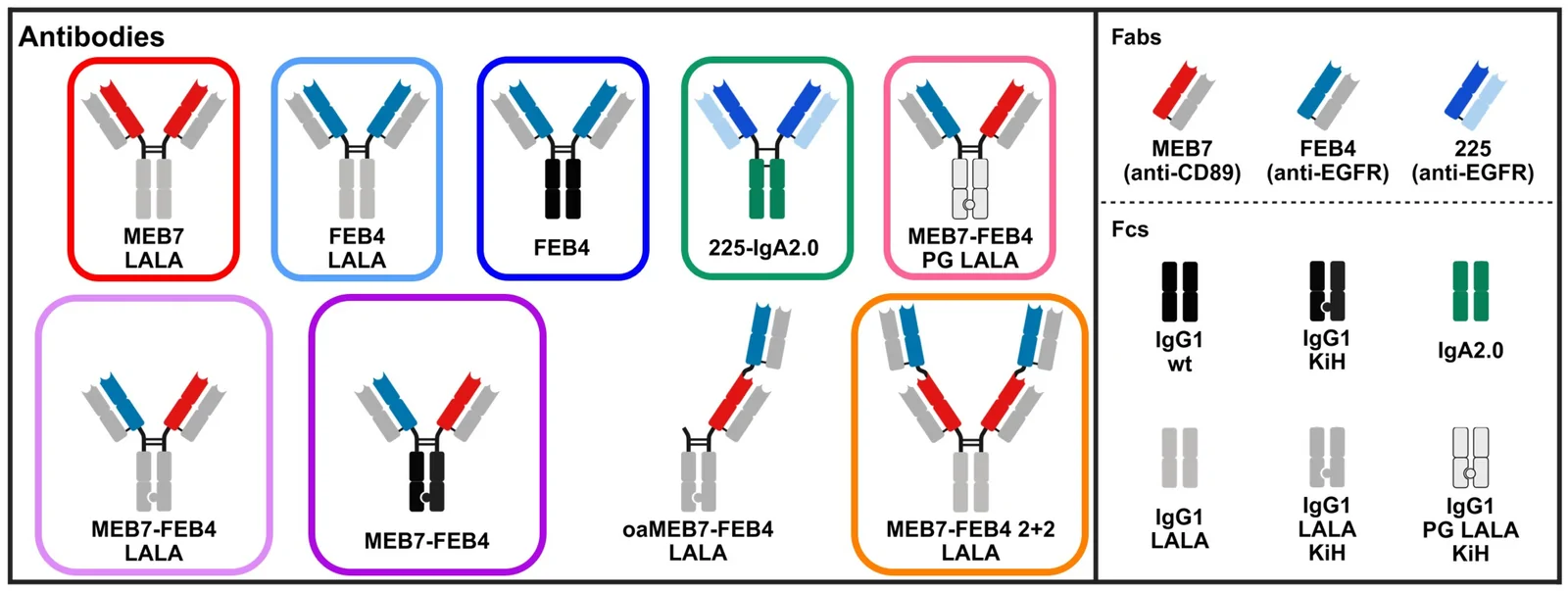
CD89 × EGFR targeting antibody constructs used in this study. The panel illustrates the Fab and Fc compositions of various antibody formats designed to engage CD89 (MEB7) and/or EGFR (FEB4, 225). Fabs are color-coded based on specificity: MEB7 (anti-CD89, red), FEB4 (anti-EGFR, blue), and 225 (anti-EGFR, light blue). Fc regions are shown with their respective engineering variants: wild-type IgG1 (black), IgG1 LALA (gray), IgG1 PG LALA (light gray), IgA2.0 (green), as well as Knobs-into-Holes (KiH). Bispecific antibodies include monovalent (MEB7-FEB4, MEB7-FEB4 LALA) and bivalent 2 + 2 formats (MEB7-FEB4 2 + 2 LALA), as well as a one-armed construct (oaMEB7-FEB4 LALA). Colors of outlines identify the antibody formats and are used consistently throughout the manuscript to distinguish the different constructs. Variants were engineered to evaluate the impact of valency and Fc modifications on myeloid-mediated tumor cell killing.

Here, we describe the isolation and functional characterization of a symmetric 2 + 2 bispecific CD89 × EGFR antibody format (Figure 1), employing a common light chain (cLC) approach to solve the light chain pairing problem while maintaining favorable manufacturability and biophysical properties. We observed that the 2 + 2 format enhanced tumor cell killing compared to monovalent constructs in vitro and that FcγR silencing improved neutrophil ADCC.

## 2. Materials and Methods

### 2.1. Origin and Identification of Chicken-Derived Antibodies

The EGFR-binding heavy chain (HC) FEB4 and the cLC dFEB1 were previously isolated and characterized, as described by Bogen et al. [16]. The anti-CD89 antibody was obtained from a yeast surface display library generated from the HC repertoire of a CD89-immunized chicken, in combination with the cLC dFEB1. The generation and screening procedure have been described in detail previously [17]. Briefly, the yeast surface display was generated using pYD1-derived vectors (Thermo Fisher Scientific, Waltham, MA, USA) for the co-expression of Fab heavy and light chain fragments under the control of the galactose-inducible promoter GAL1. Screening rounds were carried out by fluorescence-activated cell sorting (FACS) using a Sony SH800S cell sorter (Sony Biotechnology, San Jose, CA, USA). Surface Fab presentation was detected using a PE-conjugated anti-human lambda antibody (Southern Biotech, Birmingham, AL, USA), and binding to biotinylated CD89 ECD was analyzed using APC-labeled streptavidin (Thermo Fisher Scientific).

### 2.2. Vector Construction and Recombinant Protein Preparation

Codon-optimized DNA constructs encoding the extracellular domains (ECDs) of CD89 and EGFR were obtained from Twist Bioscience (South San Francisco, CA, USA) and cloned into pTT5 with a C-terminal Twin-Strep-tag. For EGFR, an additional N-terminal His-tag was included. Antigen purification was performed via Strep-tag affinity chromatography (StrepTactin XT, GE Healthcare, Chicago, IL, USA). Variable antibody domains derived from chicken immunization libraries or sourced from Twist Bioscience were grafted onto a human IgG1 framework (G1M3 allotype). The Fc-engineered IgA antibody 225-IgA2.0, described by Lohse et al. [13], was expressed from pTT5. Symmetric full-length IgG antibodies were purified using Protein A affinity chromatography (MabSelect PrismA, Cytiva, Marlborough, MA, USA). Asymmetric full-length IgGs incorporating the Knob (T366W) and Hole (T366S, L368A, Y407V) mutations were sequentially purified by IMAC (HisTrap Excel, Cytiva) via a C-terminal His-tag on the Hole HC, followed by a Strep-tag affinity purification (Twin-Strep-tag on the Knob HC; StrepTactin XT, IBA Lifesciences, Göttingen, Germany). All purified proteins were re-buffered into PBS using two consecutive HiTrap Desalting 5 mL columns (Cytiva). Effector-silenced IgGs contained L234A and L235A in the Fc region. Additional silencing was achieved by the incorporation of the P329G mutation. Stacked Fabs were connected via a partial hinge (EPKSCD) and a (G_4_S)_2_ linker. Fc-only HCs for one-armed antibodies included the hinge region up to residue C220S. All recombinant proteins were transiently expressed in Expi293F cells (Thermo Fisher Scientific) using the ExpiFectamine™ 293 Transfection Kit (Thermo Fisher Scientific) according to the manufacturer’s instructions. Purified antibodies were stored in formulation buffer at 4 °C for short-term use and at −80 °C for long-term storage. Repeated freeze–thaw cycles were avoided by storing proteins in single-use aliquots.

### 2.3. Cell Lines

A431 cells (purchased from ATCC, Manassas, VA, USA) were cultured in DMEM (Thermo Fisher Scientific) supplemented with 10% FBS (Merck Millipore, Burlington, MA, USA) and 1% penicillin–streptomycin (Sigma Aldrich, Burlington, MA, USA) at 37 °C and 5% CO_2_. THP-1 cells (purchased from ATCC) were maintained in RPMI-1640 medium (Thermo Fisher Scientific) with identical supplementation and culture conditions. Expi293F cells (purchased from Thermo Fisher Scientific) were cultured in Expi293F Expression Medium (Thermo Fisher Scientific) at 37 °C, 5% CO_2_ and agitation at 110 rpm. BHK-21 cells stably transfected with CD89 (kindly provided by the group of Prof. Dr. med. Thomas Valerius, UKSH, Kiel, Germany) were maintained in RPMI-1640 medium supplemented with 10% FBS, 1% penicillin–streptomycin, 100 µM sodium pyruvate (Gibco, Grand Island, NY, USA), and 1% non-essential amino acids (Sigma Aldrich) [18]. For selection, 125 µM methotrexate (Sigma Aldrich) and 1 mg/mL Geniticin (Carl Roth, Karlsruhe, Germany) were included in the culture medium.

### 2.4. Isolation of Human Peripheral Blood Mononuclear Cells (PBMCs) and Polymorphonuclear Cells (PMNs)

Peripheral blood (80 mL) was collected from healthy donors following informed written consent. PBMCs and PMNs were isolated from citrate-anticoagulated blood via density gradient centrifugation using Polymorphprep (Serumwerk Bernburg, Bernburg, Germany), according to the manufacturer’s instructions. Residual erythrocytes were removed by ice-cold hypotonic lysis. Cell viability, assessed by trypan blue exclusion, exceeded 95%.

### 2.5. Affinity Determination, Receptor–Ligand Competition, and Simultaneous Dual Antigen Engagement via Bio-Layer Interferometry

BLI measurements were performed using the Octet RED96 system (FortéBio, Molecular Devices, San Jose, CA, USA) at 30 °C with agitation at 1000 rpm. Biosensors were pre-equilibrated in PBS (pH 7.4) for 10 min prior to loading the antibody or antigen of interest (10 µg/mL) until a wavelength shift of 1 nm or saturation was achieved. All subsequent steps were performed in kinetics buffer (Sartorius, Göttingen, Germany).

For affinity determination of MEB7-based constructs to CD89-ECD, anti-human IgG Fc capture biosensors (AHC, Sartorius) were loaded with the antibody of interest, followed by association with serial dilutions of CD89-ECD (4.4–120 nM) for 300 s, and dissociation for 300 s. Binding kinetics were analyzed using a 1:1 Langmuir binding model with Savitzky–Golay filtering.

Receptor–ligand competition between MEB7 and IgA for CD89 was assessed using biotinylated CD89-ECD immobilized on High Precision Streptavidin biosensors (SAX, Sartorius) until saturation was reached. Application of the first antibody was followed by either a mixture of the first and second antibody or the first antibody alone, each for 1200 s. 225-IgA2.0 was applied at 500 nM and MEB7 at 50 nM.

Simultaneous dual engagement of EGFR and CD89 by bispecific antibodies was evaluated using AHC biosensors loaded with the antibody of interest. Subsequently, EGFR-ECD and CD89-ECD were applied sequentially at 50 nM for 350 s each, analogous to the competition assay.

### 2.6. Cellular Binding Assays

Cellular binding of antibodies was assessed by affinity titration. Briefly, 1 × 10^5^ cells were washed with ice-cold PBS-B (PBS supplemented with 0.5% bovine serum albumin, Carl Roth) and incubated with a serial dilution of the respective antibody for 30 min on ice. Following two additional washes with PBS-B, the cells were incubated with the appropriate secondary antibody for 15 min on ice. After a final wash, the geometric mean fluorescence intensity was measured using a CytoFLEX S flow cytometer (Beckman Coulter, Brea, CA, USA).

For IgA2.0 constructs, or if Fc interactions were expected to influence binding, light chain staining was performed using anti-human κ or λ antibody-PE conjugate (Southern Biotech). In all other cases, anti-human IgG-PE conjugate (Thermo Fisher Scientific) was used as the secondary antibody.

### 2.7. THP-1 Differentiation

THP-1 cells were centrifuged at 500× *g* and washed with PBS prior to resuspension in culture medium containing 20 ng/mL phorbol 12-myristate 13-acetate (PMA; Thermo Fisher Scientific) for 24 h. Following PMA treatment, cells were incubated in fresh medium for 48 h to allow for recovery and maturation. M1 differentiation was induced by treatment with 250 ng/mL lipopolysaccharide (LPS, *E. coli* O111: B4; Merck KGaA) for 48 h.

### 2.8. ADCP Assay

THP-1 cells were differentiated in 96-well cell culture plates (Greiner, Kremsmünster, Austria) at 6 × 10^4^ cells per well. On the day of the assay, target cells were detached using trypsin and washed with PBS. Macrophage-like THP-1 cells were stained with 5 μM Calcein Red-Orange (Sigma Aldrich) in medium and target cells with 5 μM Calcein AM (Sigma Aldrich) for 1 h at 37 °C in PBS. After staining and washing three times with PBS, target cells were resuspended in culture medium and 6 × 10^4^ cells per well were added to the M1-like cells corresponding to an effector-to-target (E:T) ratio of 1:1. After the addition of the samples, the cells were co-incubated for 4–5 h at 37 °C. After trypsinization, the samples were analyzed using a CytoFLEX S flow cytometer. Relative tumor cell uptake was determined from the Calcein AM fluorescence signal detected in THP-1 cells and expressed as fold change relative to the buffer control samples.

### 2.9. Neutrophil ADCC Assay

Antibody-dependent cellular cytotoxicity (ADCC) mediated by neutrophils was assessed using a chromium-51 (^51^Cr, Revvity, Waltham, MA, USA) release assay. Target cells (6 × 10^5^) were labeled with 50 µCi (1.85 MBq) of ^51^Cr for 2 h at 37 °C in culture medium. After thorough washing, labeled target cells were mixed with PMNs and antibodies at an E:T ratio of 40:1 in round-bottom microtiter plates, with 2 × 10^5^ PMNs per well. Granulocyte-macrophage colony-stimulating factor (GM-CSF; CellGenix, Freiburg, Germany) was added to a final concentration of 50 U/mL. Following 4 h of incubation at 37 °C, the release of ^51^Cr into the supernatant was measured in counts per minute (CPM) by mixing the samples with OptiPhase HiSafe 3 scintillation cocktail (Revvity) prior to scintillation counting (MicroBeta TriLux scintillation counter, Perkin Elmer, Turku, Finland). Specific lysis was calculated using the formula: percentage lysis = [(sample CPM − spontaneous release)/(maximum release − spontaneous release)] × 100. Spontaneous release was determined from target cells incubated with neutrophils in complete medium, while maximum release was determined from target cells treated with 5% Triton X-100 (Roche Diagnostics, Copenhagen, Denmark). For comparative analysis across donors, specific lysis values were normalized within each biological replicate to the upper plateau response of the 225-IgA2.0 positive control, which was set to 100%, and are presented as relative specific lysis.

### 2.10. Statistical Analysis

Data were analyzed using Graphpad Prism version 10.1.0. Unless otherwise indicated, data are represented as mean ± SD. Statistical significance was assessed using one-way ANOVA with appropriate post hoc multiple comparison testing as indicated in the respective figure legends.

## 3. Results

### 3.1. Identification and Characterization of Novel Anti-CD89 Antibody MEB7

**Figure 2:**
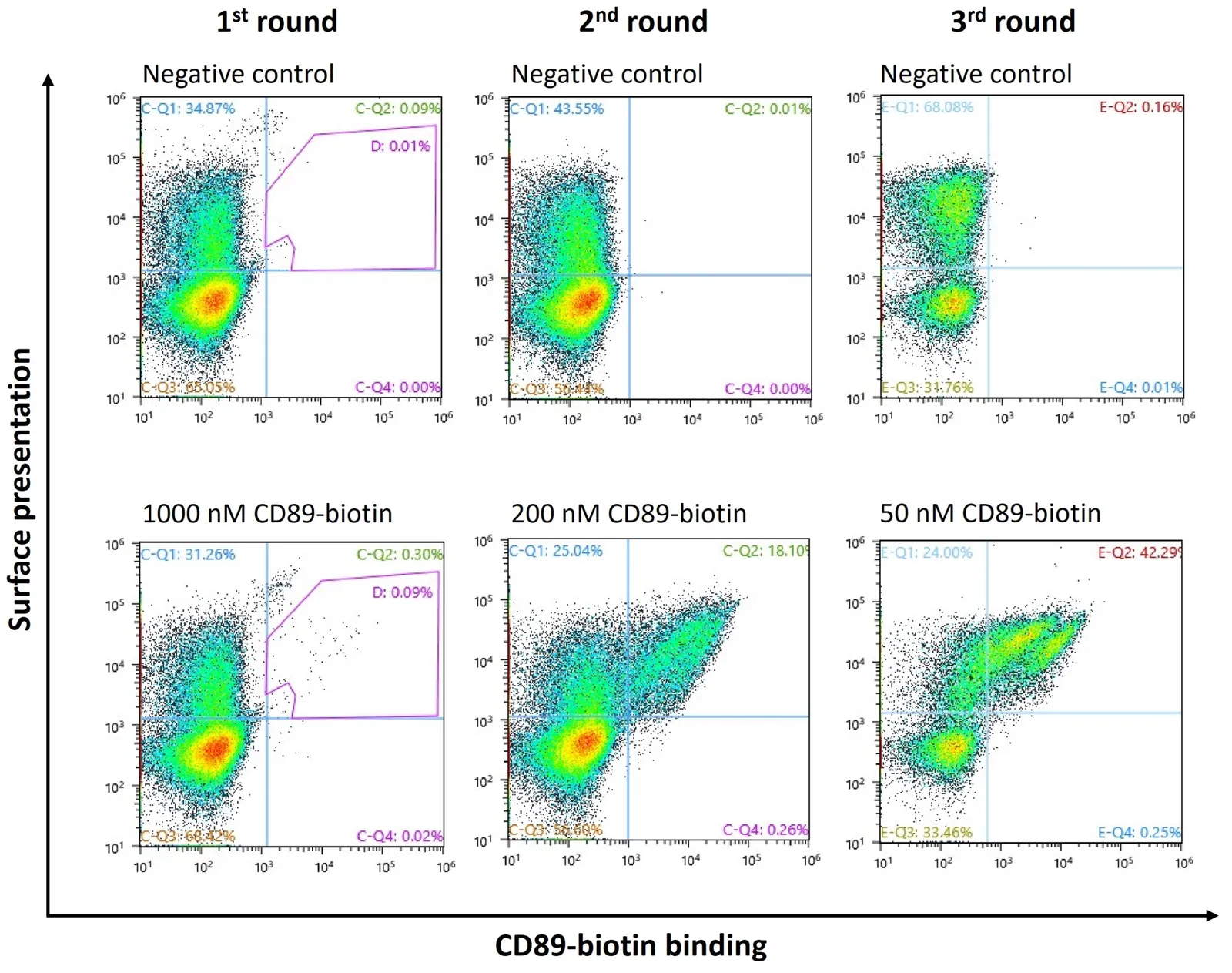
FACS-based screening of a chicken-derived CD89 Fab yeast surface display library. Surface Fab presentation was detected using a PE-conjugated anti-human lambda antibody. Binding to biotinylated CD89-ECD was assessed using APC-labeled streptavidin. Colors indicate cell density (pseudocolor). For the generation of the library, the heavy chain repertoire from a CD89-immunized chicken was combined with the common light chain dFEB1.

We aimed to isolate a CD89-binding Fab with a cLC to facilitate bispecific antibody development and avoid light chain mispairing. To this end, the cLC dFEB1, previously developed for the anti-EGFR antibody FEB4, was employed due to its favorable biophysical stability and low aggregation propensity [16]. The CD89 binding Fab MEB7 was identified through a yeast surface display screening of an immune library derived from a chicken immunized with the ECD of human CD89 (Figure 2). Briefly, the variable heavy chain repertoire from CD89-immunized chickens was paired with the dFEB1 light chain and displayed on the yeast surface. High-affinity clones were enriched over three rounds of FACS under increasing selection stringency, achieved by stepwise reduction of antigen concentration. The final high-stringency sorting round was included to enrich high-affinity binders, as the initial objective was to identify antibodies suitable for monovalent and bispecific formats with reduced avidity. Following the final sorting round, individual clones were screened, leading to the identification of MEB7 as a high-affinity CD89-binding Fab.

Notably, the EGFR-targeting Fab FEB4, which shares the cLC dFEB1 with MEB7, has previously been shown to potently inhibit ligand-induced EGFR signaling and downstream proliferative pathways [17]. The use of this shared light chain enabled the seamless generation of bispecific constructs combining MEB7 and FEB4 (Figure 1). Analytical characterization by SDS-PAGE and size-exclusion chromatography (SEC) demonstrated efficient assembly of the recombinant antibody formats (Figure S1, Table S1). In particular, the symmetric 2 + 2 bispecific construct exhibited a highly homogeneous profile with low aggregate content, comparable to that of conventional IgG-like formats.

**Figure 3:**
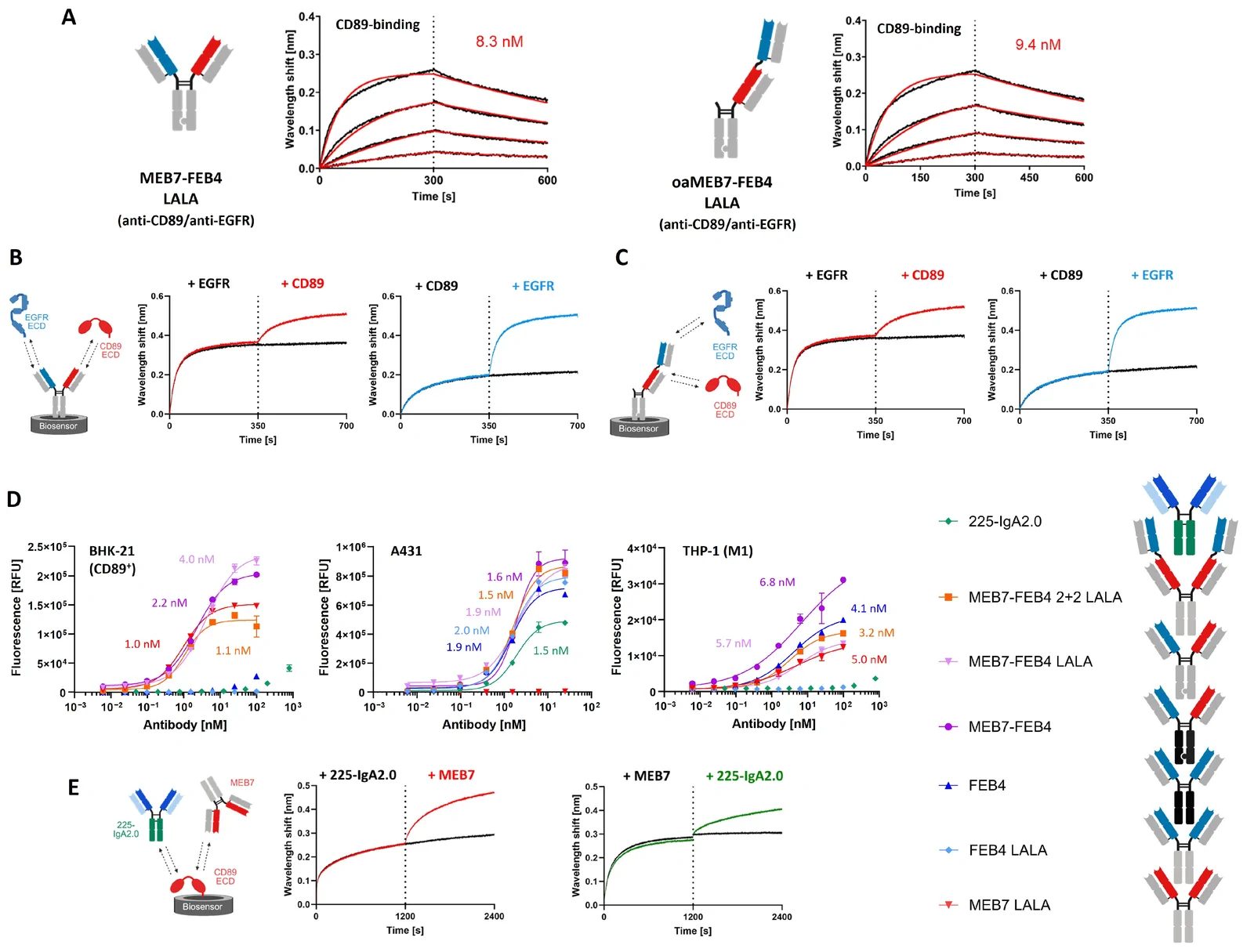
Binding characterization of MEB7-FEB4 bispecific antibodies and 225-IgA2.0. (**A**) BLI analysis of CD89 binding by MEB7 in different bispecific configurations. Antibodies were immobilized on anti-human IgG Fc Capture (AHC) biosensors and incubated with a serial dilution of CD89-ECD (4.4–120 nM). Binding kinetics were fitted using a 1:1 Langmuir model; experimental data are shown in black, fitted curves in red. Apparent dissociation constants (KDs) are indicated. (**B**,**C**) Simultaneous dual antigen engagement. One armed and two armed MEB7-FEB4 were loaded onto AHC biosensors and antigens were added sequentially (each at 50 nM) for 350 s. (**D**) Cell-based binding analysis of antibodies to CD89-expressing BHK-21 cells (**left**), EGFR-expressing A431 cells (**middle**) and THP-1-derived M1-like macrophages (**right**). On BHK-21 and A431, anti-κ light chain and anti-IgG Fc were used as the secondary antibody for 225-IgA2.0 and the remaining antibodies, respectively. On THP-1 cells anti-κ light chain was used as the secondary antibody for 225-IgA2.0. For the remaining antibodies, anti-λ light chain was utilized. EC50 values were determined using a four-parameter variable slope model and are indicated in the plot. (**E**) Competitive binding assay assessing co-engagement of CD89 by MEB7 and 225-IgA2.0. Biotinylated CD89-ECD was immobilized on High Precision Streptavidin (SAX) biosensors, followed by sequential addition of MEB7 and 225-IgA2.0 to determine potential binding interference.

Bio-layer interferometry (BLI) confirmed that MEB7 retained high-affinity binding to CD89 with K_D_ values in the single digit nanomolar range in both standard and stacked Fab bispecific formats (Figure 3A). BLI also demonstrated simultaneous binding to CD89 and EGFR by the bispecific antibodies, irrespective of format (Figure 3B,C). In cellular binding assays using BHK-21 cells stably expressing human CD89 and MEB7 exhibited low-nanomolar EC50 values (Figure 3D) in all antibody constructs. In contrast, 225-IgA2.0, a cetuximab-derived IgA2.0 antibody, did not yield a measurable EC50 under these conditions, consistent with its lower affinity for CD89 observed by BLI (~250 nM; Figure S2).

Since macrophages provide a more physiologically relevant setting than engineered BHK-21 cells, binding was additionally assessed in THP-1-derived M1-like macrophages (Figure 3D). Although this model does not capture the full phenotypic and functional heterogeneity of primary human monocyte-derived macrophages or TAMs, it provides a practical and reproducible high-throughput system for comparative evaluation [19]. All CD89-targeting constructs demonstrated high apparent affinity for macrophages (low nanomolar EC_50_ values), in line with results in the transfected cells. An improved binding response was observed with Fc-competent constructs, which was attributed to the binding of THP-1 expressed FcγRs additional to CD89 engagement. As expected, FEB4 exhibited efficient Fcγ-mediated binding, whereas the Fc-silenced FEB4 LALA variant did not. The polarization of THP-1s with LPS induced a strong upregulation of surface CD89 expression contributing to the high apparent affinity and efficacy of CD89-targeting antibodies (Figure S3). In contrast, a three- to fivefold lower affinity of these constructs to PMNs was observed (Figure S4). Notably, the maximal binding response of 225-IgA2.0 on A431 cells is not directly comparable to IgG1-based antibodies, as a different secondary detection antibody was required for this format.

To investigate epitope competition, a BLI-based epitope binning experiment was performed using MEB7 and 225-IgA2.0, which targets the D1 domain of CD89. Both antibodies bound immobilized CD89 but showed reduced secondary binding, which was attributed to steric hindrance between the antibodies while binding to partially overlapping or adjacent epitopes (Figure 3E).

### 3.2. CD89 Engagers Potently Activate Myeloid Effector Responses

**Figure 4:**
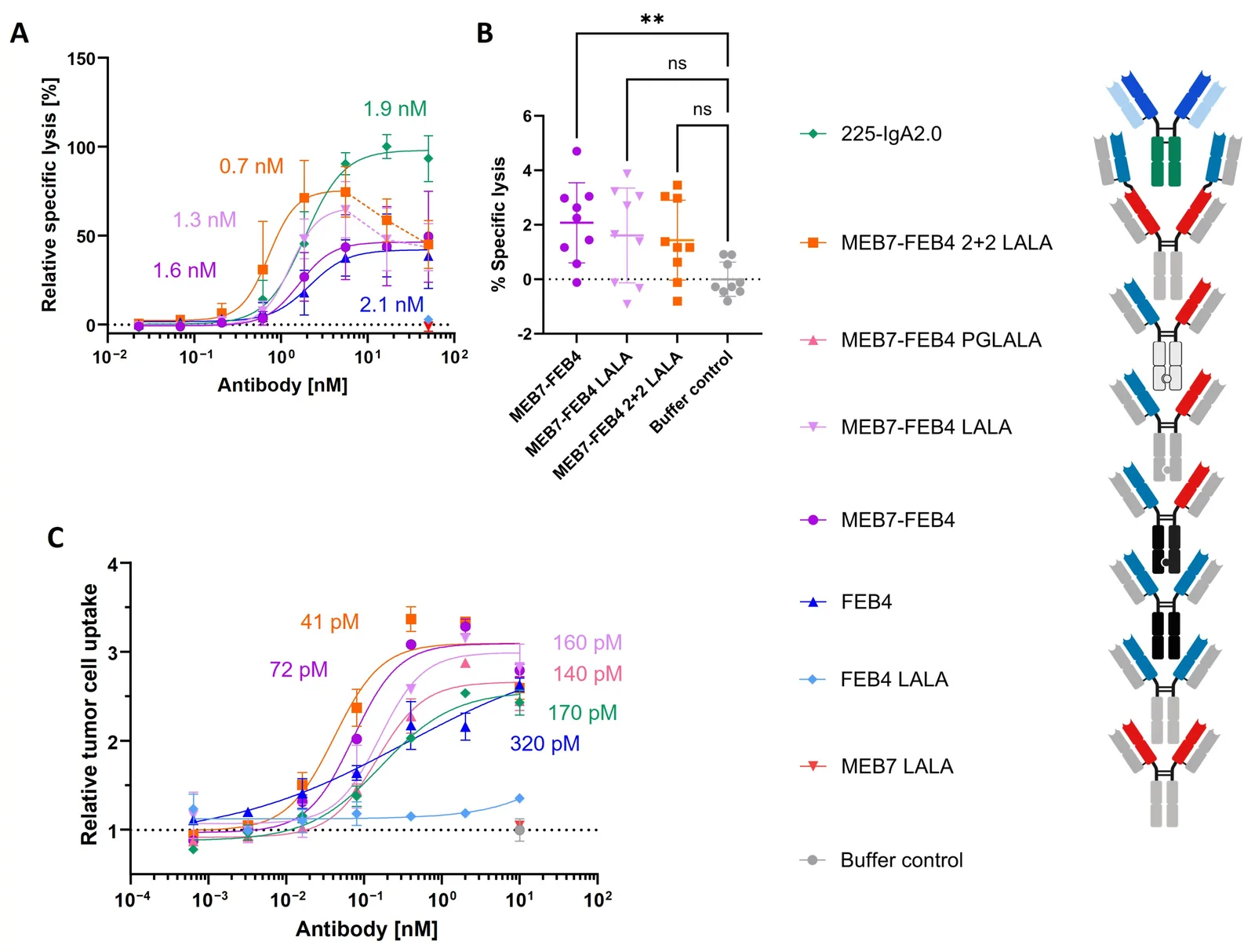
CD89 × EGFR-targeting antibodies induce PMN-mediated cytotoxicity and macrophage phagocytosis. (**A**,**B**) For ^51^Cr release assays, effector cells and ^51^Cr-labeled target cells were co-incubated at an E:T ratio of 40:1 for 4 h in the presence of 50 U/mL GM-CSF, before radioluminescence of the supernatant was measured. Specific lysis was calculated relative to spontaneous and maximal release controls and normalized within each donor to the upper plateau response of 225-IgA2.0. (**A**) Antibody concentration-dependent PMN-mediated ADCC against A431 cells. (**B**) PMN-mediated fratricide of PMNs in the presence of 15 nM antibody. Data points beyond the maximal response, consistent with saturation or hook effects, are connected by dotted lines; curve fitting was restricted to the ascending portion of each curve. (**C**) THP-1 cells were differentiated with phorbol 12-myristate 13-acetate (20 ng/mL, 24 h), rested for 48 h, and polarized with lipopolysaccharide (250 ng/mL, 48 h) to generate M1-like macrophages. Macrophages were labeled with Calcein Red-Orange and A431 target cells with Calcein AM, then co-incubated with antibody at an E:T ratio of 1:1 for 4–5 h. Following detachment, samples were analyzed by flow cytometry, and phagocytosis was quantified as uptake of Calcein AM-positive target material by THP-1 cells, expressed as tumor cell uptake relative to buffer controls. Data were fitted using a four-parameter logistic model, and EC50 values are indicated. Data in (**A**,**B**) represent at least *N* = 3 biological replicates with *n* = 3 technical replicates. (**C**) shows one representative experiment of three independent experiments performed in duplicate, with comparable results. Error bars indicate SD. ns: *p* > 0.05; **: *p* < 0.01; (one-way ANOVA with Dunnett’s multiple comparisons test).

As neutrophils are key CD89-expressing effector cells, we first evaluated neutrophil-mediated ADCC using freshly isolated human PMNs (Figure 4A). Consistent with previous reports showing superior neutrophil activation by IgA antibodies, 225-IgA2.0 mediated stronger ADCC than the FcγR-engaging IgG1 antibody FEB4 [20]. Notably, the Fc-silenced MEB7-FEB4 LALA variant showed enhanced ADCC activity over much of the tested concentration range compared with its Fc-competent counterpart, suggesting that reduced FcγR engagement can enhance CD89-driven PMN cytotoxicity. Among the Fc-silenced constructs, the 2 + 2 bispecific format displayed approximately a twofold lower EC_50_ than the bispecific bivalent variant, reaching sub-nanomolar potency. Both constructs exhibited a hook effect at higher antibody concentrations, as commonly observed for multispecific engagers when excess antibody limits productive cell–cell crosslinking.

Potential antibody-induced neutrophil fratricide was assessed by quantifying specific lysis in co-cultures of labeled PMNs with activated PMNs (Figure 4B). Overall, specific lysis remained low under the conditions tested, although a slight increase was observed for all constructs relative to the buffer control. Statistical significance was reached only for the wild-type Fc-bearing CD89 engager MEB7/FEB4. However, the absolute magnitude of this effect was modest. These data indicate that increased CD89 valency did not provoke pronounced neutrophil-mediated killing of immune cells at the tested concentration. No significant PMN-mediated fratricide of PBMCs was observed (Figure S5). Likewise, a complementary fratricide assay using PBMCs as effectors (Figure S6) revealed no detectable cytotoxicity induced by CD89-bivalent or Fc-competent antibodies under the conditions tested.

In addition to neutrophils, macrophages constitute a major myeloid effector population in solid tumors and can mediate antitumor activity through phagocytosis of opsonized target cells and subsequent antigen presentation. We therefore evaluated whether CD89-targeting antibodies also promote macrophage-mediated tumor cell uptake. Antibody-dependent cellular phagocytosis (ADCP) was assessed using a flow cytometry-based assay quantifying uptake of labeled A431 target material by THP-1-derived M1-like macrophages (Figure 4C).

Here, CD89-targeting bispecific antibodies mediated higher maximal ADCP responses than the IgA comparator 225-IgA2.0, indicating efficient induction of macrophage tumor cell uptake. Across all formats, CD89-targeting bispecific antibodies robustly induced macrophage phagocytosis, with EC50 values in the low triple-digit picomolar range. The 2 + 2 LALA variant demonstrated a three- to fourfold increase in potency compared with the monovalent LALA-silenced construct while achieving comparable maximal phagocytic responses. To further distinguish Fc-mediated contributions from CD89-specific activity, a 1 + 1 PGLALA construct was included because residual activity was observed for the LALA-silenced variant. ADCP activity of the 1 + 1 formats generally followed their degree of Fc competence, with the wild-type IgG1 variant showing higher maximal responses and improved potency compared with the PGLALA construct, while the LALA variant displayed an intermediate profile.

## 4. Discussion

The engineering of immune cell engagers that effectively harness myeloid effector functions represents a promising strategy for cancer immunotherapy, particularly for solid tumors in which T cell infiltration is limited. In this study, we developed and characterized EGFR × CD89 bispecific antibodies designed to redirect neutrophils and macrophages towards tumor cells. Using a symmetric 2 + 2 bispecific format in combination with LALA Fc silencing modification, we identified a construct that mediated potent cytotoxic and phagocytic activity across both effector cell populations.

The LALA mutation was incorporated to strongly attenuate complement activation and FcγR-mediated off-target interactions, including the potential fratricide among CD89-positive myeloid cells [21]. Although no pronounced fratricide was observed for the 2 + 2 construct under the in vitro conditions tested, these assays do not exclude delayed or indirect toxicities arising from sustained myeloid cell activation, cytokine release, or systemic inflammation. Comprehensive safety assessment will therefore require dedicated in vivo studies, which were beyond the scope of the present work.

In our phagocytosis assays, LALA silenced constructs displayed minimal Fc-driven activity on their own. However, constructs simultaneously targeting CD89 consistently showed a modest increase in efficacy compared with the PGLALA variants. This observation suggests that residual IgG1 Fc activity may become functionally relevant during productive CD89 co-engagement.

In contrast to the enhancing effect of Fc competence observed in macrophage phagocytosis, neutrophils possess a distinct Fcγ receptor repertoire that may differentially shape antibody-mediated responses. Neutrophils predominantly express FcγRIIIb (CD16b) together with lower levels of FcγRIIa (CD32a), whereas expression of high-affinity FcγRI (CD64) is minimal under resting conditions [22]. Although CD16b lacks a classical ITAM signaling domain, FcγR engagement can modulate neutrophil activation, and blockade of CD16b has been reported to enhance IgG1-mediated ADCC [20,23]. This may help explain why the FcγR-silenced MEB7-FEB4 LALA variant mediated stronger PMN cytotoxicity than its FcγR-competent counterpart.

The hook effect observed for the LALA constructs likely reflects reduced productive effector–target cell bridging at high antibody concentrations, a phenomenon commonly seen with immune cell engagers. Consistent with this, recent modeling studies identified engager concentration, arm affinity, and receptor density as key determinants of bridge formation [24]. Tuning the relative affinities of the tumor- and effector-binding arms may therefore broaden the active concentration window, although excessive reduction of CD89 affinity could compromise potency.

While our in vitro assays with surrogate M1-like macrophages and GM-CSF-stimulated neutrophils provide initial functional and mechanistic insights, they do not fully reflect the immunosuppressive microenvironment of advanced solid tumors, where complex myeloid phenotypes arise and frequently promote tumor growth [25]. Within the TME, CD89 expression can be downregulated on monocyte-derived macrophages, reducing the efficacy of antibodies against it [26]. Additionally, excessive antigen display following phagocytosis may potentially contribute to T cell exhaustion [27]. Nonetheless, prior work by Xu et al. demonstrated that CD89/HER2 and CD89/CD20 bispecific antibodies induce antitumor responses in checkpoint-resistant tumors in CD89 transgenic mice [10]. Their efficacy was attributed to the conversion of M2 to M1 macrophages and enhanced antigen presentation, leading to improved CD8^+^ T cell responses. In neutrophils, IgA- but not IgG-engagement has been shown to trigger LTB4 secretion and neutrophil swarming, suggesting that CD89 targeting can have beneficial effects even within immunosuppressive tumor microenvironments [9]. These effects may be further amplified by combination strategies that promote M2-to-M1 repolarization, such as toll-like receptor agonists or innate immune checkpoint inhibitors, or through integration with an established checkpoint blockade [28,29].

Overall, our findings highlight CD89 as a promising target for engaging myeloid effector cells in solid tumors. LALA-modified EGFR × CD89 bispecific antibodies enabled potent activation of both neutrophils and macrophages while reducing the liabilities associated with wild-type FcγR engagement. While the 2 + 2 architecture may require further optimization with respect to size and tumor penetration, these in vitro data support the further development of CD89-directed bispecific antibodies as myeloid-targeted cancer immunotherapies. As this study focused on the in vitro characterization of the engineered antibody format, future studies in appropriate CD89 transgenic mouse models will be required to evaluate its antitumor efficacy and safety in vivo.

## Data Availability

The raw data supporting the conclusions of this article will be made available by the authors on request.

## Supplementary Materials

The following supporting information can be downloaded at: https://www.mdpi.com/article/10.3390/antib15040066/s1, Figure S1: Reducing SDS-PAGE of antibodies used in this study; Table S1: SEC analysis of antibodies used in this study; Figure S2: BLI analysis of the interaction of 225-IgA2.0 with immobilized CD89; Figure S3: FcγR and FcαRI expression of THP-1 cells in dependence on differentiation; Figure S4: Cellular binding of CD89/EGFR bispecific antibodies to human polymorphonuclear cells (PMNs) freshly isolated from peripheral blood; Figure S5: PMN-mediated fratricide of peripheral blood mononuclear cells PBMCs measured by ^51^Cr release; Figure S6: PBMC-mediated killing of THP-1 derived M1-like macrophages.

## Abbreviations

The following abbreviations are used in this manuscript:

ADCC	Antibody-dependent cellular cytotoxicity
ADCP	Antibody-dependent cellular phagocytosis
AHC	Anti-human IgG Fc capture biosensors
APC	Allophycocyanin
BLI	Bio-layer interferometry
cLC	Common light chain
CPM	Counts per minute
E:T	Effector-to-target ratio
ECD	Extracellular domain
EC50	Half maximal effective concentration
EGFR	Epidermal growth factor receptor
Fab	Fragment antigen-binding
FACS	Fluorescence-activated cell sorting
FcRn	Neonatal Fc receptor
FcγR	Fc gamma receptor
FcαRI	Fc alpha receptor I (CD89)
GM-CSF	Granulocyte-macrophage colony-stimulating factor
HC	Heavy chain
Ig	Immunoglobulin
IL	Interleukin
IMAC	Immobilized metal affinity chromatography
ITAM	Immunoreceptor tyrosine-based activation motif
K_D_	Equilibrium dissociation constant
KiH	Knobs-into-Holes
LALA	Fc-silencing mutation containing L234A and L235A substitutions
LPS	Lipopolysaccharide
LTB4	Leukotriene B4
PBMC	Peripheral blood mononuclear cell
PBS	Phosphate-buffered saline
PBS-B	PBS supplemented with bovine serum albumin
PE	Phycoerythrin
PMA	Phorbol 12-myristate 13-acetate
PMN	Polymorphonuclear cell
PGLALA	Fc-silencing mutation containing P329G, L234A, and L235A substitutions
SAX	Streptavidin biosensors
SEC	Size-exclusion chromatography
TAM	Tumor-associated macrophage
TAN	Tumor-associated neutrophil
TME	Tumor microenvironment
TGF	Transforming growth factor
^51^Cr	Chromium-51
