# Supplementary material for: Fc-Silenced CD89 × EGFR Bispecific Antibodies Promote Neutrophil and Macrophage Antitumor Activity
Source: Antibodies (Basel). 2026 Jul 29;15(4):66. doi: 10.3390/antib15040066 (PMC13509766; doi:10.3390/antib15040066)
Supplement: Supplementary file 1 [file antibodies-15-00066-s001.zip › antibodies-4363933-supplementary.pdf]

Supplementary Material

Fc-silenced CD89 × EGFR bispecific antibodies promote neutrophil and macrophage antitumor activity

Felix Meiser, Julia Harwardt, Christoph Hahn, Marta Lustig, Thomas Valerius, Harald Kolmar

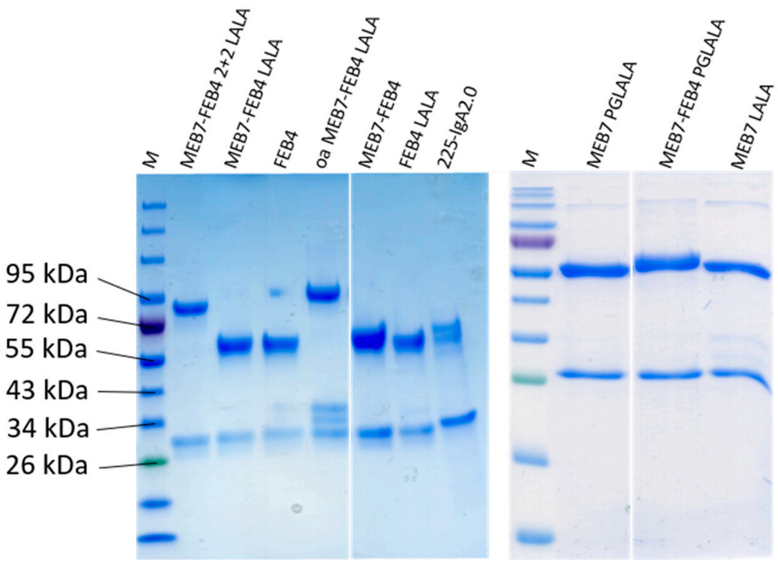

Figure S1: Reducing SDS-PAGE of antibodies used in this study. The Color Prestained Protein Standard, Broad Range was used as molecular weight standard (M). The gel was stained with Coomassie Brilliant Blue staining solution. 4 µg of protein were loaded onto the gel for each sample. The major bands correspond to the stacked heavy chain (HC; ~75 kDa), HC (~50 kDa), truncated HC (~25 kDa), and light chain (~22 kDa). An additional HC band is presumed to represent a putative glycoform.

Table S1: SEC analysis of antibodies used in this study. To analyze aggregation and fragmentation of antibodies, SEC was performed using the Size Exclusion (SW-Type) HPLC Column TSKgel® in an HPLC 1260 infinity device. For each analysis 100 µL sample containing 30 µg antibody were loaded onto the column. Chromatography was performed at a flow rate of 0.35 mL/min for 20 min and protein was detected by measuring the absorbance at 280 nm.

Antibody	% Aggregated	% Monomeric
MEB7 LALA	1.3	98
FEB4 LALA	2.5	97
FEB4	0.6	99
225-IgA2.0	0.8	83
MEB7-FEB4 LALA	3.5	85
MEB7-FEB4	1.7	95
oaMEB7-FEB4 LALA	0	83
MEB7-FEB4 2+2 LALA	0	100
MEB7-FEB4 PG LALA	2.1	96
MEB7 PGLALA	1.4	98

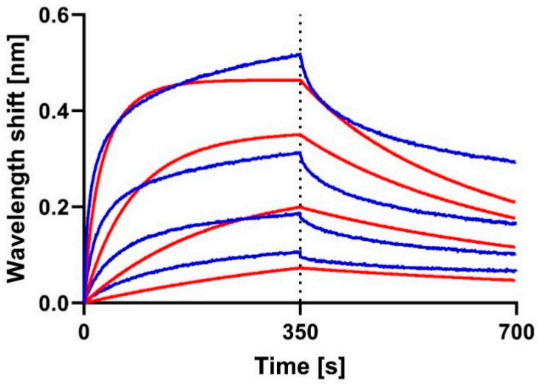

Figure S2: BLI analysis of the interaction of 225-IgA2.0 with immobilized CD89. CD89-biotin was immobilized on a SAX biosensor and the biolayer increase upon sequential association of 225-IgA2.0 at different concentrations (1024-16 nM) was measured. Biosensor data was fitted utilizing a 2:1 binding model. The biosensor signal is shown in blue and the fit in red. The apparent K_D was approximately 250 nM.

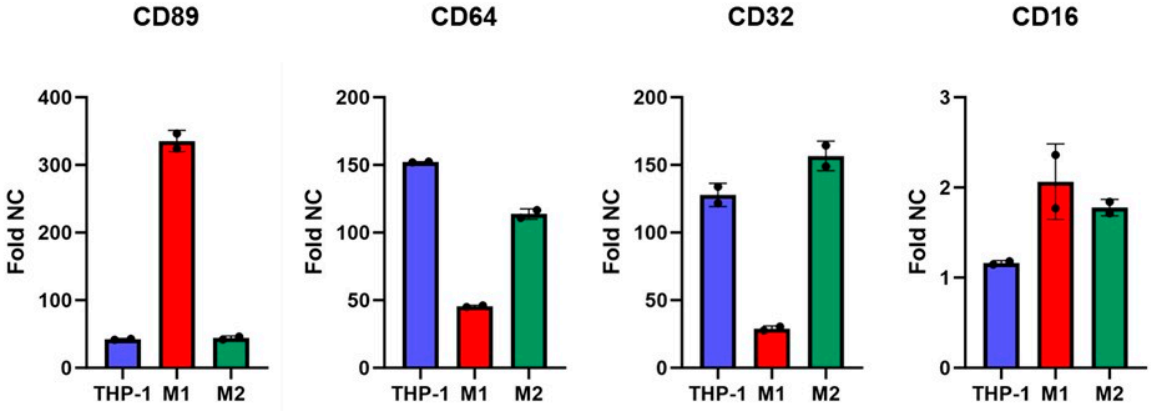

Figure S3: FcγR and FcαRI expression of THP-1 cells in dependence on differentiation. For M2 differentiation, THP-1 cells were treated with 5 ng/mL PMA for one day before letting them rest in medium for two days. The cells were then polarized to M2 macrophages by the addition of 20 ng/mL IL-4 (Thermo scientific) and IL-13 (Acro Biosystems) to the medium. To differentiate into M1, 20 ng/mL PMA and 250 ng/mL LPS were used instead. CD89 was stained with MEB7 PGLALA and anti-human IgG-Fc-PE conjugate. CD64 and CD16 were stained with commercial antibody-APC conjugates (Sino Biological, BioLegend), and CD32 with an antibody-PE conjugate (Sino Biological). Unstained controls of each phenotype were subtracted respectively divided by the negative control of THP-1 in the respective channel to calculate Fold NC.

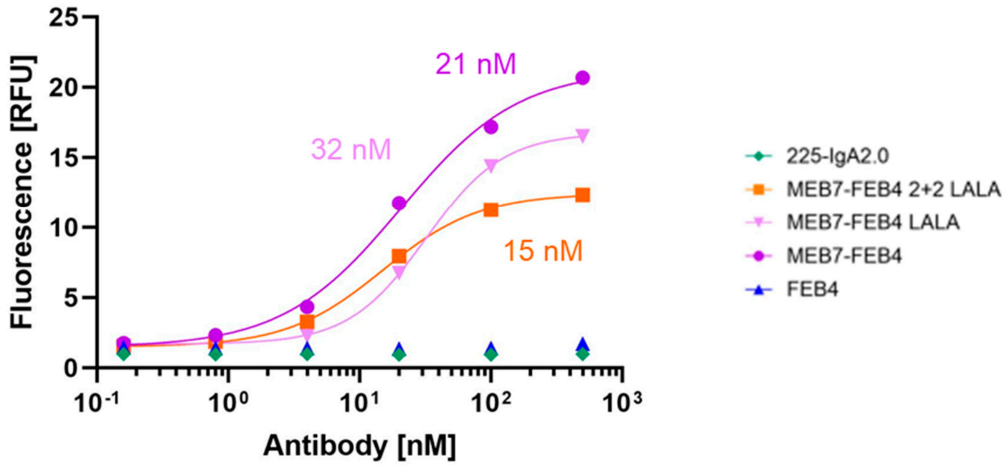

Figure S4: Cellular binding of CD89/EGFR bispecific antibodies to human polymorphonuclear cells (PMNs) freshly isolated from peripheral blood.
Data were fitted using a four-parameter logistic model, and EC50 values are indicated.

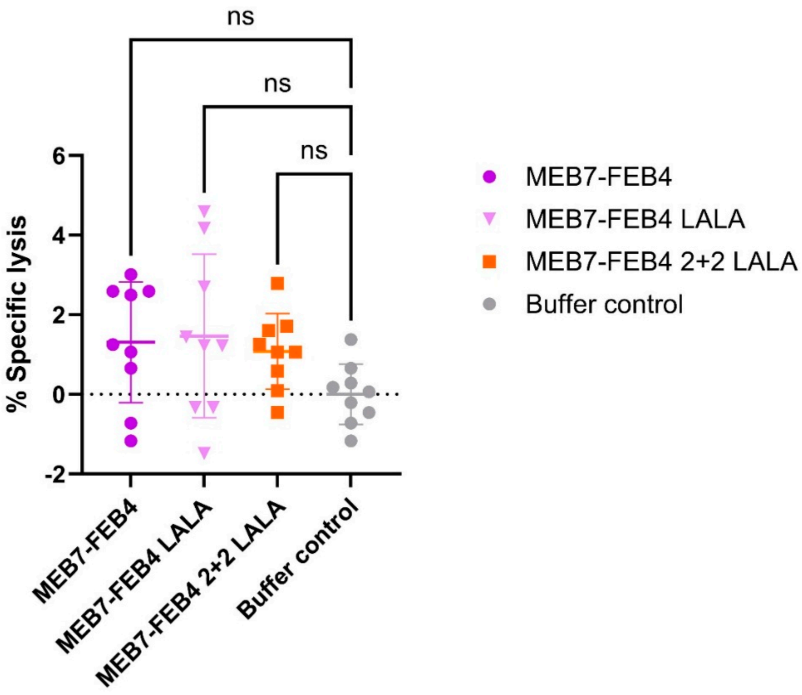

Figure S5: PMN-mediated fratricide of peripheral blood mononuclear cells PBMCs measured by 51Cr release.
Effector and labeled target cells were co-incubated at an E:T ratio of 40:1 for 4h in the presence of 15 nM antibody before measuring radioluminescence of the supernatant. Data represent N = 3 biological replicates with n = 3 technical replicates; error bars indicate SD. ns > 0.05, * p < 0.05 ** p < 0.01 *** p < 0.001, by one-way ANOVA with Dunnett's multiple comparisons test.

PBMC-mediated fratricide of THP-1-derived M1-like macrophages
Buffy coats of healthy donors from the German Red Cross Blood Donation Service Baden-Württemberg-Hessen, Frankfurt am Main, Germany were kindly provided by the working group of Evelyn Ullrich (Department of Pediatrics, Goethe University Frankfurt, Frankfurt am Main, Germany). PBMCs were isolated by density gradient centrifugation, as described by Graafen et al. (Immune profiling and functional analysis of NK and T cells in ataxia telangiectasia). The PBMC-mediated killing assay was based on the protocol described by Yamashita et al. (A novel method for evaluating antibody-dependent cell-mediated cytotoxicity by flow cytometry using cryopreserved human peripheral blood mononuclear cells). In short, THP-1 cells were differentiated to M1-like macrophages by sequential PMA (20ng/mL) and LPS (250 ng/mL) treatment leading to the upregulation of CD89 expression. The THP-1 cells were then labeled with 5 µM carboxyfluorescein succinimidyl ester (CFSE, eBioscience) for 5 minutes. After washing the cells three times in PBS they were resuspended in growth medium and seeded into 96-well F-bottom culture plates before incubation for 24 hours at 37°C, and 5% CO_2. The next day, PBMCs were thawed and added to the target cells at an E:T ratio of 5:1. Antibodies were added in RPMI 1640 supplemented with 10% heat-inactivated FBS and 1% PenStrep. After 24 h co-incubation at 37°C, and 5% CO_2, cells were harvested by trypsinization. The positive control was incubated with 10% (v/v) of PBS + 1% Triton X-100 (Sigma Aldrich) for 3 min at 37°C. Cells were washed once with PBS, followed by staining with Fixable Viability Dye (FVD, eBioscience) for 20 minutes on ice in the dark. After two more washes with PBS, cells were resuspended in PBS and analyzed via CytoFLEX S. FVD (APC-A750 channel) signal of CFSE-positive target cells (FITC channel) was analyzed.

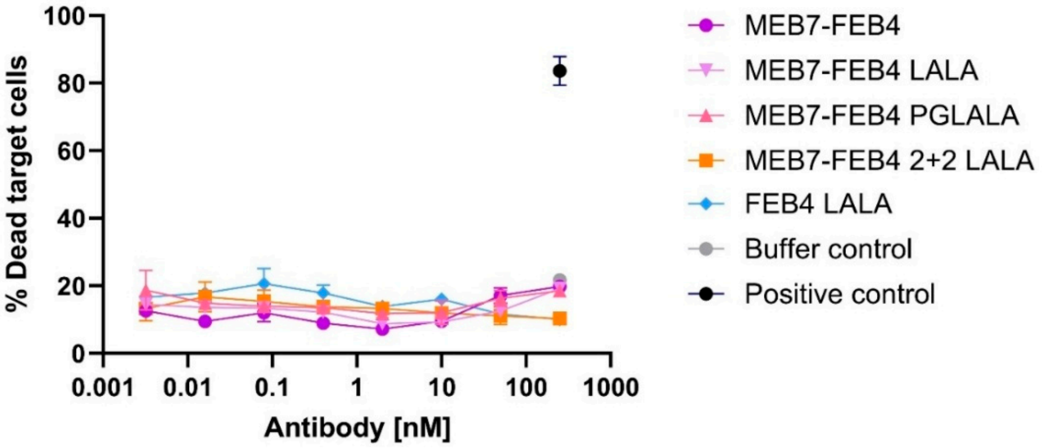

Figure S6: PBMC-mediated killing of THP-1 derived M1-like macrophages. Percentage of dead target cells represents the ratio of FVD/CFSE-double positive to all CFSE-positive cells. PBMCs and M1-like macrophages were co-incubated with antibody for 24h at an E:T ratio of 5:1. Positive control was treated with 0.1% Triton X-100. Error bars indicate the standard deviation of experimental duplicates.
